# Benign breast lesions in Bayelsa State, Niger Delta Nigeria: a 5 year multicentre histopathological audit

**DOI:** 10.11604/pamj.2014.19.394.5717

**Published:** 2014-12-18

**Authors:** Stanley Chibuzo Uwaezuoke, Ezenwa Patrick Udoye

**Affiliations:** 1 Department of Surgery, Federal Medical Centre, Yenagoa, Bayelsa State, Nigeria; 2 Department of Anatomical Pathology, Niger Delta University Teaching Hospital, Okolobiri, Bayelsa State, Nigeria

**Keywords:** Benign breast disease, lesions, fibroadenoma, fibrocystic change

## Abstract

**Introduction:**

There has been no previous study to classify benign breast lesions in details based on histopathologically confirmed diagnosis in Bayelsa State, Nigeria. This study therefore aims to review all cases of benign breast lesions seen in all the three centres in Bayelsa State with histopathology services over a five year period for a comprehensive baseline data in our community for management, research and education.

**Methods:**

This is a multicentre retrospective descriptive study based on histopathological diagnosed benign breast lesions from January 2009 to December 2013. Archival results and slides on benign breast lesions were retrieved and analysed using simple statistical methods.

**Results:**

A total of 228 benign breast lesions (68.3%) were seen among 334 histopathologically diagnosed breast diseases. The male to female ratio was 19.7:1. Peak age incidence was the third decade (43%) with a mean age of 29.1years. Fibroadenoma was the most common benign breast disease (BBD) accounting for 45.6% of all the cases followed by fibrocystic change (23.1%). The mean ages of fibroadenoma and fibrocystic change were 23.1years and 31.1years respectively. Inflammatory breast lesions constituted 8.3%. We recorded only 2 cases (0.9%) of atypical ductal hyperplasia (ADH) with no case of atypical lobular hyperplasia (ALH) within the study period. Gynaecomastia (4%) was the main male breast lesion in the study.

**Conclusion:**

Benign breast diseases are the most common breast lesions in Bayelsa State. Fibroadenoma is the most common lesion followed by fibrocystic change. The incidence of atypical hyperplasia recorded was rather low in the state.

## Introduction

The human breast is paired mammary glands composed of specialized epithelium and stroma in which can occur both benign and malignant lesions [1]. Benign breast diseases (BBD) however constitute the greater of the breast lesions [2–6]. These BBD are diverse, ranging from disorders of development, inflammatory lesions, proliferative diseases of the epithelium and stroma to different types of neoplasms [1, 7]. Though most of the available literature show that breast lumps are mostly benign and non-proliferative epithelial lesions, it is known that certain benign breast diseases (BBD) are important risk factors for breast cancers which can develop in either breast later [8, 9]. Also literature has shown that proliferative breast diseases without atypia have a relative risk (risk compared to women without any risk factors) of 1.5 to 2.0 of developing invasive carcinoma while proliferative breast diseases with atypia (ADH and ALH) poses a 4.0 to 5.0 relative risk of invasive cancer development [1]. Other researchers equally have placed the relative risk of invasive cancer development at 1.3 to 1.9 for proliferative breast diseases without atypia [10–12]. Dupont et al however stated a relative risk of 3.1 for atypical lobular hyperplasia [11]. Over the years, in Nigeria there has been an increasing awareness of breast cancer with its attendant morbidity and mortality amongst the general populace, particularly from organized programmes by the Federal and State Ministries of Health, Non-Governmental Organizations (NGOs), the various medical bodies, religious organizations, media outlets and most especially medical publications. This increased awareness has also been associated with much anxiety in persons who notice lumps in their breasts, necessitating increased presentations at health facilities. There is a great need for surgeons, pathologists, radiologists and oncologists not only to recognize benign breast lesions but to have a baseline data to aid accurate diagnosis, treatment, prognostication and ability to access prevalence of such lesions at risk of malignant transformation in our local environment. This multicentre analysis, a pioneer study in Bayelsa State of Nigeria aims at auditing the benign breast lesions in our environment to provide a comprehensive data that will form the basis for diagnosis, treatment, research and education.

## Methods

This is a multicentre retrospective descriptive study based on all the surgical breast specimens received at the only three histopathology services centres in Bayelsa State including Federal Medical Centre Yenagoa, Niger Delta University Teaching Hospital, Okolobiri and a Private Histopathology facility in Yenagoa all in Bayelsa State, Nigeria from January 2009-December 2013. All the original request forms and histopathological reports on the breast specimens received within this study period in these three centres with their slides were retrieved from the archives and reviewed. From the request forms and histopathological reports, information on the age, sex, nature of specimen, hospital numbers, laboratory numbers and histopathological diagnosis were extracted. New slides were made from formalin fixed, paraffin-embedded tissue blocks and stained with Haematoxylin and Eosin (H&E) where necessary for appropriate diagnosis and classification. The results obtained were analyzed using simple descriptive statistical methods. Cases of breast lesions with incomplete data and cases of which we were unable to trace their slides or blocks were excluded from the study.

## Results

Out of 334 histopathologically diagnosed breast lesions, 228 (68.3%) were benign in nature. The ratio of benign to malignant lesions was 2.2:1. Of these 228 benign breast lesions seen, 217 (95.2%) were in females while 11 (4.8%) were in males giving a female to male ratio of 19.7:1. Table 1 shows Age and Sex distribution of benign breast lesions seen in the study. The age range of benign breast lesions was 14-67years while the peak incidence age range was the third decade (98; 43%). The mean age of the benign breast lesions was 29.1years. Fibroadenoma was the most common BBD as well as the most common fibroepithelial tumour encountered in the study; accounting for 104 cases (45.6%) of the total (Table 2). Fibroadenoma occurred most within the second and third decades of life in this study (Table 3) with a mean age of 23.1years. Fibrocystic change formed the second most common benign breast lesion accounting for 60 (26.3%) of all cases, occurring most in the third and fourth decade of life (Table 4) with a mean age of 31.1years. Inflammatory lesions of the breast accounted for 19 (8.3%) (Table 2) of the total benign breast lesion with most occurring within the 4th and 5th decades of life. These included fat necrosis 6, chronic non-specific mastitis 5, breast abscesses 4, chronic granulomatous lesions 3, duct ectasia 1. A few lesions of the breast skin were seen, 9 in number of which 6 were epidermal cysts, 2 fibroepithelial polyps and a keloid. Two cases of atypical ductal hyperplasia were observed in this study. No case of lobular hyperplasia with atypia was seen. Out of the 11 benign breast diseases found in males in this study, 9 (81.8%) were Gynaecomastia. Others were 1 lipoma and 1 epidermal cyst. The age range of occurrence of Gynaecomastia in this study was 16-70years.

## Discussion

Generally BBD constitute about 90% of breast lesions across the globe [13, 14]. The results of the index study showed that benign breast lesions constituted 68.3% (228) of the total breast lesions (334) studied. This is similar to the findings in other series in Nigeria: in which 67% [15], 68.8% [2], 71.2% [16], 72.5% [1], 73% [17] and 73.4%^18^ were seen in Markudi, Enugu, Warri, Benin, Kano and Calabar respectively. On the other hand, lower figure of 59.5% [19] BBD was seen in Gombe while much higher figures of 87% [20] and 89% [1] were recorded in Ilesha and Ibadan respectively. In Tikrit city Iraq, BBD constituted 70.3% of total breast lesions in figures which is similar to our finding [21]. The overall mean age of occurrence of BBDs in this study was 29.1years. Similar mean ages for BBDs were reported by Olu-Eddo et al [8] (27.5years) and Echejoh et al [15] (32.2 years). These figures show that though generally BDD are the predominant breast lesions, there are some variations in their actual incidence from place to place.

**Table 1:** Age and sex distribution of benign breast lesions

Age Range	Female	Male	Total	Percent (%)
11-20	53	1	54	23.7
21-20	95	3	98	43.0
31-40	42	3	45	19.7
41-50	17	0	17	7.5
51-60	7	2	9	3.9
61-70	3	2	5	2.2
Total	217	11	228	100

**Table 2:** Frequency distribution of benign breast lesions

Histological Types	Frequency	Percent (%)
Fibroadenoma	104	45.6
Fibrocystic change	60	26.3
Inflammatory lesions	19	8.3
Atypical Ductal Hyperplasia	2	0.9
Gynaecomastia	9	4.0
Lipoma	6	2.6
Epidermal cyst	6	2.6
Tubular adenoma	5	2.2
Lactating adenoma	5	2.2
Sclerosing adenosis	3	1.4
Benign phylloides	2	0.9
Lactational changes	2	0.9
Fibroepithelial polyp	2	0.9
Intraductal papilloma	1	0.4
Granular cell tumour	1	0.4
Keloid	1	0.4
Total	228	100.00

**Table 3:** Age distribution of fibroepithelial/stromal lesions

Age in years	11-20	21-30	31-40	41-50	51-60	61-70	Total
FIBROEPITHELIAL							
Fibroadenoma	42	49	11	2	-	-	104
Benign Phylloides	1	-	1	-	-	-	2
STROMAL							
Lipoma	-	1	1	1	2	1	6
Granular cell tumour	1	-	-	-	-	-	1

**Table 4:** Age distribution of epithelial lesions

Age in years	11-20	21-30	31-40	41-50	51-60	61-70	Total
Fibrocytic change	7	30	15	3	4	1	60
Gynaecomastia	1	3	2	-	1	2	9
Tubular adenoma	1	3	1	-	-	-	5
Lactating adenoma	-	2	3	-	-	-	5
Intraductal papilloma	-	1	-	-	-	-	1
Sclerosing adenosis	-	2	-	1	-	-	3
Lactational changes	-	1	1	-	-	-	2
Atypical ductal hyperplasia	-	1	1	-	-	-	2
Total	9	43	23	4	5	3	87

The present study showed that fibroadenoma was the most common BBD in Bayelsa State as it constituted 45.6% of all the benign breast lesions seen. This result is not surprising as most of such studies in our country showed similar findings notably in Enugu (44%) [2], Benin (43%) [8], Markudi (45%) [15], Ilesha (46.2%) [20], Port Harcourt (51%) [22] and Ife (59.1%) [23]. In Ghana, another West African state, fibroadenoma formed the most common BBD with a large frequency of 70% of the total benign lesions [24]. Abhijit et al also reported fibroadenoma as the most common breast disease forming 56.4% of the total benign breast lesions in rural India [25]. A study in Kano, Northern Nigeria however reported fibrocystic change as the most common BBD with fibroadenoma as the second [8, 16]. Also Memon et al [26] in Pakistan and Shirley et al [27] in Jamaica both reported fibrocystic change as the most common BBD in their series while another study in Karachi, Pakistan reported fibroadenoma as the most common BBD [28]. Our finding of high frequency of fibroadenoma among BBD is in concordance with most African studies and this may not be unconnected with the already noted racial predilection of blacks to the disease [28]. The index study also noted a sharp increase in the second decade with a peak in the 3rd decade for fibroadenoma followed by a drastic fall in incidence. This is in tandem with the findings of other researches in our environment and outside the country [8, 15, 21]. The mean age of occurrence of fibroadenoma among women in our study was 23.1years. This is very similar to the mean ages for fibroadenoma in Ibadan (24.4years) and Benin (22.9years) studies [3, 8].

Fibrocystic change was the second most common benign breast lesion in the present study accounting for 26.3% of the total BBD seen (Table 2). This finding is in line with most studies in Nigeria [2, 3, 8, 15, 19, 20, 22, 23]. Also other studies outside Nigeria, reported fibrocystic change as the second after fibroadenoma [29]. In a series from Northern Nigeria, fibrocystic change emerged the most common BBD [17]. Also certain studies in Iraq [21], Pakistan [26], USA [30] and Italy [31] documented fibrocystic change as the most common BBD. Our study result shows that 75% of fibrocystic change occurred in the 3rd and 4th decades of life with a mean age of occurrence of 31.1years. This agrees with similar findings by other authors in Benin [8] and Ibadan of mean ages 30years and 39.5years respectively. This result therefore supports the belief that fibrocystic change affects the older females more when compared to fibroadenoma. Inflammatory lesions seen in this study constituted 8.3% of the total BBD. This is similar to the 8.1% for inflammatory breast lesions found in Benin [8]. In Warri, Kano and Ife, inflammatory breast lesions accounted for 6.4%, 6% and 4.6% of the total BBD respectively [16, 17, 23]. However in Calabar and Port Harcourt, the authors recorded much higher frequencies of inflammatory breast lesions [18, 22]. Our figure of 8.3% may not be truly representative of the actual frequency in our local environment because many breast abscesses and other inflammatory breast conditions are managed in our hospitals and clinics with incision and drainage and antibiotics. Only a few unresolved and suspicious cases achieve histopathological consultation.

The index study recorded only two cases (0.9%) of atypical ductal hyperplasia and no case of atypical lobular hyperplasia was documented. This finding is relatively low compared to those of nearby Warri [16] and Benin^8^ where 3% and 3.9% respectively for both atypical and lobular hyperplasia were seen. Atypical epithelial hyperplasia is of great clinical significance because of its relationship with carcinoma [32]. Dupont et al [11] reported a four-fold increased risk compared to the general population for atypical hyperpasia. A relative risk of 4.0 to 5.0 have also been documented for subsequent development of invasive cancer [33]. Our current low incidence of atypical hyperplasia can be explained partly by the fact that awareness and early presentation of patients with breast lumps is still relatively low in our locale. Other benign breast diseases encountered in this study include Tubular adenoma, Sclerosing adenosis and Benign phylloides tumour. Their frequencies were low and they exhibited no peculiar age relationship. Eleven (11) males had benign breast lesions in this study of which nine (9) were Gynaecomastia constituting 4% of the total benign breast diseases seen. The other two (2) lesions were epidermal cyst and lipoma. This is very similar to the findings in Kano and Ife of 4% and 3.9% for Gynaecomastia [17, 23]. Lower figures of 2.1% and 0.9% have been recorded in Benin and Warri [8, 16]. Higher figure of 14% for Gynaecomastia was recorded in Port Harcourt [22]. Skin lesions seen in this study were predominantly epidermal cysts which is similar to the study in Benin in which the predominant lumps in the skin were epithelial cysts. Though these skin lesions are not definitive breast lesions, the elucidation of their true histologic nature helped allay the patient's anxiety.

## Conclusion

Benign breast diseases are the most common breast lesions in Bayelsa State, Nigeria. Fibroadenoma is the most common type of benign breast disease followed by fibrocystic change in our environment. Atypical hyperplasia is comparatively in Bayelsa State, Nigeria. Increased advocacy and availability of adequate screening facilities including mammography and histopathological services will go a long way towards detecting and treating atypical lesions early thereby reducing the chances of progression of these lesions to invasive cancer.
